# Role of Rosiglitazone as a Gastroprotective Agent Against Indomethacin-Induced Gastric Mucosal Injury in Rats

**DOI:** 10.4021/gr2009.12.1328

**Published:** 2009-11-20

**Authors:** Ashraf Taye, Adel H. Saad

**Affiliations:** a Department of Pharmacology and Toxicology, Faculty of Pharmacy, Minia University, Egypt; b Department of physiology, Faculty of Medicine, Minia University, Egypt

**Keywords:** Rosiglitazone, Gastric ulcer, Indomethacin, TNF-α, Lipid peroxides, Nitric oxide

## Abstract

**Background:**

Rosiglitazone, an insulin sensitizing agent, has been recently implicated in the control of inflammatory processes and modulation of expression of various cytokines such as tumor necrosis factor (TNF-α). However, its mechanistic effect of gastric mucosal integrity remains to be elucidated.

**Methods:**

The present study was designed to determine effect of rosiglitazone on gastric mucosal lesions induced by indomethacin (IND) in rats. Pyloric ligation was performed for the collection of gastric juice, and gastric ulceration was induced by a single intraperitoneal injection of IND (30 mg/kg).

**Results:**

IND administration caused a significant decrease in the volume of gastric juice mucin and gastric mucosal nitrite and prostaglandin E_2_ (PGE_2_) levels. This was accompanied by a significant increase in gastric juice free and total acidity and pepsin activity. In addition, an elevation in the gastric mucosal lipid peroxide and serum TNF-α level was observed. Pretreatment with rosiglitazone (10 mg/kg, orally, for 1 weeks) resulted in a significant reduction in the elevated gastric mucosal lesions and lipid peroxides levels. This was associated with a marked increase in gastric juice mucin and a reduction in TNF-α level. Moreover, rosiglitazone significantly increased the gastric mucosal total nitrite and PGE_2_ levels.

**Conclusions:**

Rosiglitazone exerts a gastroprotective effect against IND-induced gastric mucosal lesions and its anti-ulcer effect is mediated via scavenging free radicals, increasing NO, PGE_2_ and mucus production in addition to its anti-inflammatory mechanisms. Thus, rosiglitazone could be a relevant drug for patients taking non-steroidal anti-inflammatory drugs (NSAIDs) and at high risk of developing gastric ulceration.

## Introduction

The link between non-steroidal anti-inflammatory drugs (NSAIDs) and the presence of upper gastrointestinal complications has been well established [1, 2]. Indomethacin (IND), a potent NSAID, was introduced in 1963 for the treatment of rheumatoid arthritis and related diseases. A reduction in the biosynthesis of prostaglandin (PG) through inhibition of cyclooxygenase (COX) is the pharmacological background to both the anti-inflammatory action and the harmful side effects of IND and other NSAIDs [3]. The gastrointestinal adverse effects of NSAIDs, especially in the stomach, are one of the more serious complications in patients taking these drugs [4]. Indeed, IND shows a potent ulcerogenic action in experimental animals [5]. The mechanism by which IND induces gastric injury is generally considered to involve depletion of PGs, yet it has proven to be more complicated and involves multiple, closely interacting elements such as gastric hypermotility, microcirculatory disturbances, neutrophil-endothelial cell interactions and superoxide radicals, in addition to PG deficiency [6, 7].

The development of a novel class of insulin-sensitizing drugs, thiazolidinediones, may be considered a significant advance in anti-diabetic therapy. One key mechanism by which theses drugs exert their effects is by activation of the peroxisome proliferator-activated receptor gamma (PPAR-γ), a member of the nuclear receptors family [8]. Recent data suggest that the agonists of these receptors might also have therapeutic potential in the treatment of inflammatory diseases and certain cancers [9].

Rosiglitazone has been recently implicated in the control of inflammatory processes and in the modulation of the expression of various cytokines such as tumor necrosis factor alpha (TNF-α) [10, 11]. It has also been shown that rosiglitazone exerts a protective effect against ischemia reperfusion injury in a variety of tissues including the lung [12], the heart [13], and the brain [14]. Furthermore, rosiglitazone has proved its potential effectiveness in treatment of active ulcerative colitis via its anti-inflammatory and antioxidant effects [15]. However its role in stress induced gastric mucosal injury has not been fully emphasized.

The aim of this study was focused on investigation the possible protective effects of rosiglitazone on IND-induced gastric mucosal lesions in adult male rats and the underlying mechanism(s) involved in this setting.

## Materials and Methods

### Animals

Male Wister rats from the local strain weighing 150 – 200 g were used. That species was selected due to consistency and reproducibility of gastric ulcer model in it [16]. Rats were housed at room temperature with 12:12 h light/dark cycles. All experiments were performed during the same time of the day to avoid variations due to diurnal rhythm of putative regulators of gastric function. Experiments were conducted in accordance with the guidelines for animal care of the United States Naval Medical Research Centre, Unit No. 3, Abbaseya, Cairo, Egypt, accredited by the Association for Assessment and Accreditation of Laboratory Animal Care international (AAALAC international).

### Chemicals

Indomethacin (IND) and rosiglitazone (Rosi) were purached from Sigma Aldrich (USA).

### Pyloric ligation

All rats were fasted for 24 hours before being subjected to pyloric ligation in mesh bottomed cages to minimize coprophagy, with free access to water except for the last hour before the procedure, rats were deprived of water. Pyloric ligation was carried out in each rat under light ether anesthesia according to the method previously described [17].

### Experimental groups

After pyloric ligation, rats were divided randomly into three experimental groups of 8 rats each. 1, control group, in which rats were left freely wandering in their cages for 3 hours after receiving a single intraperitoneal (IP) injection of 1% aqueous solution of Tween 80 (vehicle of IND). 2, IND group, in which gastric ulceration was induced by a single IP injection of IND (30 mg/Kg) [18]. 3, Rosi + IND group, in which rats were given rosiglitazone (10 mg/kg, IP) for 7 successive days and then gastric ulceration was induced by IND [19].

Three hours after IND administration, blood samples were taken from the heart under ether anesthesia before rats were sacrificed by an ether overdose. Their stomachs were removed, opened along the greater curvature and the gastric content of each stomach was collected. The stomachs were washed with ice-cold saline and examined for gross gastric mucosal lesions using a magnified lens.

### Assessment of gastric mucosal lesions

Gastric mucosal lesions were examined using a magnified lens. The severity of the lesions was expressed in terms of the ulcer index (U.I.) [20]. The lesions were scored as follows: 1 for small petechiae and 2-5 for lesions of 2-5 mm length. The sum of the total scores in each group divided by the number of animals was expressed as the mean U.I. for that group.

### Analysis of the gastric juice

The gastric juice collected from each stomach was centrifuged at 1000 g for 10 minutes to remove any solid debris and the volume of the supernatant was measured. The supernatant was then analyzed for the determination of free and total acid outputs, pepsin and mucin concentrations.

### Determination of free and total acidity of the gastric juice

The free acidity was determined by titration of a given volume of the gastric juice against 0.1N sodium hydroxide up to 5.5 as guided by a pH meter. The total acidity which is composed of both mineral and organic combined acids in the gastric juice was determined by completing the titration in the above procedure for determining free acidity to pH 7 as guided by the pH meter [21].

### Determination of the proteolytic activity

The pepsin activity is the major factor involved in the proteolytic activity of gastric secretion. It was determined by a modified spectrophotometric method as previously described [22].

### Colorimetric assay for mucins and glycoproteins in gastric juice

It is a sensitive and specific method for saccharides, which is linked via N-acetylgalactosamine through O-glycosidic linkage to serine/threonine in mucins. The method is not affected by the carbohydrates present in other types of glycoproteins [23].

### Biochemical analysis of gastric mucosa

The stomach of each rat was divided into two parts: one part was immersed in IND (10 µg/ml) and was immediately stored at –80°C. Subsequently, the gastric mucosa was scraped, homogenized in 2 ml normal saline containing 0.1 M dithiothreitol and centrifuged at 2000 g for 10 minutes at room temperature. The supernatant was analyzed for determination of prostaglandin content. The mucosa of the other part of the stomach was also scraped, homogenized in cold potassium phosphate buffer (0.05 M, pH 7.4) and centrifuged at 5000 g for 10 minutes at 4°C. The supernatant was kept at –80°C for subsequent measurement of lipid peroxides and Nitric oxide. Total protein concentration was also determined using a bicinchoninic acid (BCA) protein assay kit (Pierce Chemicals).

### Determination of gastric mucosal prostaglandin E_2_

Prostaglandin E_2_ (PGE_2_) in the gastric mucosa was determined by enzyme-linked immunosorbent assay (ELISA) using PGE_2_ assay kit (R&D Systems, USA) and based on the competitive binding technique in which PGE_2_ present in a sample competes with a fixed amount of horseradish peroxidase (HRP)-labeled PGE_2_ for sites on a mouse monoclonal antibody [24].

### Determination of gastric mucosal nitric oxide

Gastric mucosal nitric oxide (NO) was determined using commercially available kits for the Colorimetric determination of total nitrite (Biodiagnostic, Egypt) and based on the enzymatic conversion of nitrate to nitrite by nitrate reductase. The reaction is followed by a colorimetric detection of nitrite as an azo dye product of the Griess [25].

### Determination of gastric mucosal lipid peroxides

Malondialdehyde (MDA) levels in the gastric mucosa were determined as an indicator of lipid peroxidation by thiobarbituric acid method as previously described [26].

### Determination of serum TNF-α level

Serum TNF-α concentration was measured in this study by enzyme-linked immunosorbent assay (ELISA) using rat TNF-α assay kit (Biosource, USA) following the instructions of the manufacturer and based on previously described method [27].

### Statistical analysis

Data were expressed as mean ± standard error of the mean (SEM). For comparison between the two means, unpaired Student’s t-test and ANOVA for multiple comparisons were used. P value less than 0.05 was considered statistical significance. Statistical analysis was performed using GraphPad Prism 5 (USA).

## Results

### Effect of rosiglitazone on gastric juice parameters

Table 1 shows that IND administration caused significant decrease in the volume of gastric juice and mucin concentration, which was accompanied by significant increase in gastric juice free and total acidity and pepsin activity. Pretreatment with rosiglitazone increased significantly gastric juice mucin concentration, but it failed to produce any significant change in gastric juice free and total acidity or pepsin activity compared to IND group.

**Table 1:** Effect of IND on Gastric Juice Parameters and Their Alteration by Rosiglitazone

Groups	Volume (ml/3h)	FAO (mEq/3h)	TAO (mEq/3h)	Pepsin activity (mg/ml)	Mucin Content (mg% hexose)
Control	2.33 ± 0.23	46.75 ± 2.82	81.61 ± 3.28	3.14 ± 0.23	79.7 ± 4.05
IND	0.81 ± 0.1^*^	91.47 ± 4.14^*^	101.75 ± 4.68	8.07 ± 0.35^*^	27.26 ± 2.13^*^
IND + Rosi	0.8 ± 0.11	87.39 ± 2.71	91.93 ± 4.95	7.11 ± 0.66	69.16 ± 2.07^**^

Data represent the mean ± SEM of observations from 8 rats. ^*^ P < 0.05 significantly different of IND-treated group versus control and IND + Rosi groups; ^**^ P < 0.01 significantly different of IND + Rosi versus IND. IND: indomethacin; Rosi: rosiglitazone; Total acid outputs; TAO; Free acid outputs, FAO.

### Effect of IND on the development of gastric mucosal lesions and its alterations by rosiglitazone pretreatment

**Figure 1:**
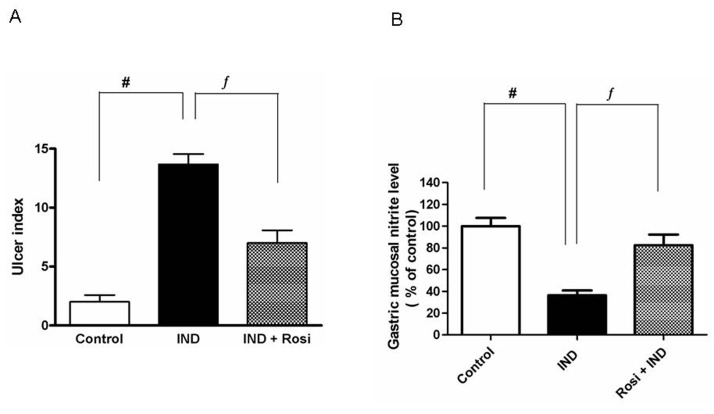
Effect of IND on the development of gastric mucosal lesions and gastric mucosal nitrite level. A, Effect of IND on the development of gastric mucosal lesions. Results are expressed as mean ± SEM of 8 rats. # is significantly different from control group; f significantly different from IND at P < 0.01 and P < 0.05, respectively. B, Effect of IND on gastric mucosal nitrite level. Results are expressed as mean ± SEM of observations from 8 rats (data are in pg/mg wet tissue normalized and expressed as % of control). # is significantly different from control group; ¦ is significantly different from IND at P < 0.01 and P < 0.05, respectively. Symbols as in Table 1.

Figure 1A shows that IND markedly (P < 0.01) induced a high ulcer index, reaching to about 3-fold of the control group. However, rosiglitazone pretreatment profoundly (P < 0.01) attenuated the ulcerative lesions and decreased the ulcer index.

### Measurement of the gastric mucosal nitrite level in IND-induced gastric ulcer

IND significantly (P < 0.01) lowered the gastric mucosal nitrite level to one-third of the control level. Pretreatment with rosiglitazone markedly increased the gastric mucosal nitrite level reaching approximately to the normal control level (Fig. 1B).

### Effect of rosiglitazone on gastric mucosal lipid peroxides

**Figure 2:**
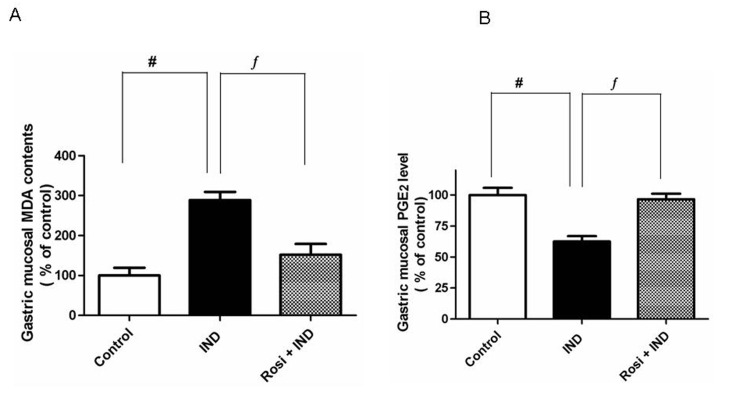
Effect of IND on gastric mucosal malondialdehyde (MDA) and gastric mucosal PGE_2_ levels. A, Effect of IND on gastric mucosal MDA level. Results are expressed as mean ± SEM of observations from 8 rats (data are in pg/mg wet tissue normalized and expressed as % of control). # is significantly different from control group; f is significantly different from IND at P < 0.001 and P < 0.05, respectively. B, Effect of IND on gastric mucosal PGE_2_ level. Results are expressed as mean ± SEM of observations from 8 rats (data are in pg/mg wet tissue normalized and expressed as % of control). # is significantly different from control group; f is significantly different from IND at P < 0.01 and P < 0.05, respectively. Symbols as in Table 1.

IND treatment significantly (P < 0.001) elevated the gastric mucosal MDA concentration (as a biochemical marker of lipid peroxidation). There was 3-fold increase in MDA contents in IND-treated rats compared to control. Pretreatment with rosiglitazone resulted in a significant reduction in the gastric MDA level compared to IND-treated group (Fig. 2A).

### Determination of the gastric mucosal PGE_2_ level

As illustrated, IND significantly (P < 0.01) lowered the gastric mucosal PGE_2_ concentrations compared to one-third of the control group. However, rosiglitazone was able to restore the attenuated level of gastric PGE_2_ almost to the control level (Fig. 2B).

### Measurement of the serum TNF-α level

**Figure 3:**
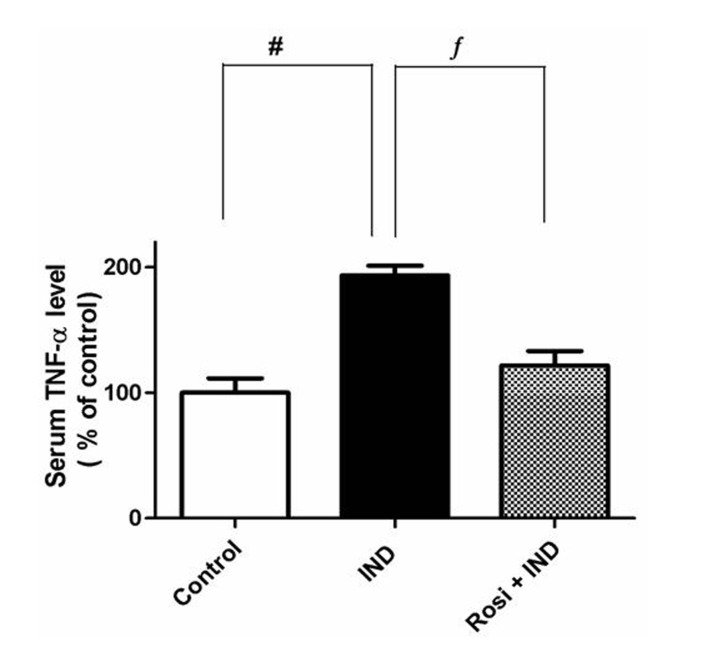
Effect of IND on the serum TNF-α level. Results are expressed as mean ± SEM of observations from 8 rats (data are in pg/mL normalized and expressed as % of control). # is significantly different from control group; f is significantly different from IND at P < 0.01 and P < 0.05, respectively. Symbols as in Table 1.

To verify the anti-inflammatory effect of rosiglitazone on IND-induced gastric ulcer, we measured the serum level of TNF-α. Although IND significantly (P < 0.01) increased the serum TNF-α level, about 2-fold compared to that of the control group (pg/mL), rosiglitazone reduced the elevated TNF-α level near to the normal level (Fig. 3).

## Discussion

Recently, the prescription of combined drugs has become extremely challenging. NSAIDs are one of the most widely used classes of drugs in the world. NSAID-induced gastric ulceration is the major side effect of this kind of drugs [28]. Therefore, drugs that have the potential to reduce NSAIDs side effects should be selected for patients taking NSAIDs concomitantly for treatment of other medical conditions [28].

Rosiglitazone is an antidiabetic drug in the thiazolidinedione class drugs [29]. It acts via activation of the intracellular receptor class of PPAR-γ [8]. Apart from its effect on insulin resistance, rosiglitazone appears to have an anti-inflammatory and antioxidant effects in a variety of inflammatory conditions including the gut [11, 14, 30]. Therefore, this study was an attempt to investigate the possible gastroprotective effect of rosiglitazone on IND-induced gastric ulcer in rats. If proved so, rosiglitazone could have an advantage over other antidiabetic drugs by providing those patients additional protection against gastric ulceration if they are at high risk due to concomitant administration of NSAIDs.

In the present study, IND administration induced severe gastric mucosal ulcerations, which were accompanied by significant increase in gastric acidity, pepsin activity, MDA and TNF-α with concomitant reduction in NO, mucin and PGE_2_ levels compared to the control rats. The gastrotoxic effects of NSAIDs, including IND are attributed to the non-selective inhibition of cyclooxygenases (COX1 and COX2) with subsequent reduction in PGs production, which are believed to have potent anti-ulcer and cytoprotective properties [2, 4, 31]. Ulceration due to NSAIDs could also be due to their ability to induce reactive oxygen metabolites, which may intern promote lipid peroxidation and gastric damage [32].

In the current study, rosiglitazone pretreatment reduced significantly the ulcerative lesions induced by IND, which were associated with significant decrease in both lipid peroxides and TNF-α level together with concomitant increase in NO, PGE_2_ and mucin levels compared to non-treated IND group. These results suggest that the protective effect of rosiglitazone may have multiple components in its actions.

Free radicals production has been reported to play a fundamental role in the pathogenesis of NSAIDs-induced gastric damage [33]. In the present study, the toxic effects of these reactive oxygen species (ROS) were evidenced by significant increase in MDA levels, which was associated with the provocation of ulcerative lesions. Rosiglitazone pretreatment significantly decreased lipid peroxides levels, which was accompanied with marked attenuation in the gastric lesions compared to non-treated IND group. Rosiglitazone was reported to enhance the expression of antioxidant enzymes namely xanthine oxidase and superoxide dismutase [34]. This increase in the antioxidant activity, in turn counteracts the deleterious effects of ROS with subsequent attenuation of mucosal damage. Therefore, the antioxidant property of rosiglitazone could be a part of its protective effect against IND-induced gastric ulceration.

NSAIDs could be proinflammatory by increasing TNF-α [35], which was confirmed in the present study. IND administration markedly increased the plasma level of the TNF-α, this effect was reversed by rosiglitazone pretreatment. TNF-α is a potent stimulator of neutrophil infiltration and plays a crucial role in the progression of ulcer injury via production of the injurious ROS [36]. The reduction in TNF-α by rosiglitazone, in turn inhibits neutrophil infiltration with subsequent oxidative burst of reactive oxygen species resulting in attenuation of the ulcerative lesions [37]. Previous studies reported that rosiglitazone exerts a potent an anti-inflammatory effect by inhibiting the expression of TNF-α in a variety of tissues including the stomach [34, 38]. Therefore, these findings provide an additional evidence for the gastroprotective effects of rosiglitazone against IND-induced gastric ulceration, which could be mediated by its anti-inflammatory action via inhibition of inflammatory cytokines (e.g. TNF-α) production as well as inhibition of ROS production.

NO plays a critical role in modulating several components of gastric mucosal defense including gastric mucosal blood flow, neutrophil adhesion and mucus secretion [39], thus affording gastric protection. Earlier studies revealed that endogenous NO released from vascular endothelium, sensory nerves or gastric epithelium cooperates with endogenous prostaglandins in the maintenance of gastric mucosa integrity and microcirculation [40].

IND administration significantly decreased the tissue nitrite level compared to control group, which was associated with ulcerative lesions. Since NO is the endothelium derived relaxing factor, reduction its level might contribute to reduce mucosal blood flow by the vasoconstriction response with subsequent gastric damage [41]. Rosiglitazone pretreatment markedly increased the NO level in gastric mucosa that resulted in attenuation of gastric lesions. Recent studies have shown that PPAR-γ agonist pioglitazone (same class as rosiglitazone) increases NO production and enhances ulcer healing, this effect was abolished by pretreatment of L-NNA, an NO synthase inhibitor [42]. These findings suggest that rosiglitazone could have similar stimulatory effect on NO, which was confirmed in our study. The increased NO level by Rosiglitazone could be attributed to the activation of NO synthase by phosphorylation and increase NO bioavailability [43]. Based on these findings, increased production of NO could be a potential target for the gastroprotective effect of rosiglitazone in this study.

Inhibition of gastric prostaglandin (PGs) synthesis is central to the ability of NSAIDs to cause gastric damage [2]. Subsequently, agents that interfere with the ability of NSAIDs to suppress gastric PGs synthesis will reduce the ability of those agents to cause damage. In the present study, IND administration caused a marked reduction in PGE_2_ level, which was associated with the development of gastric ulceration. Rosiglitazone pretreatment reversed the condition and increased significantly the PGE_2_ level with significant attenuation in the ulcerative lesions, compared to the non-treated IND group. Previous studies have shown that the rosiglitazone-induced prostaglandin production could be mediated by influences at the level of both cyclo-oxygenase-2 expression and substrate formation [44] Another possible mechanism for increased PGE_2_ level may be explained in part by the stimulatory effect of rosiglitazone on NO production [43]. It was reported that NO increased PGE_2_ synthesis in vivo through cGMP-independent mechanism and it was assumed that NO might regulate the release and/or the synthesis of PGE_2_ in the stomach after damage [45].

The secretion of mucus, one of the several defensive factors in the gastrointestinal tract [46] is another possible target for the action of rosiglitazone. IND caused a significant reduction in mucus secretion, which was reversed by rosiglitazone. Mucus secretion is physiologically regulated by both NO [47] and PGE_2_ [42], and since both NO and PGE_2_ were significantly increased by rosiglitazone, it will be expected to find a concomitant increase in mucus level with subsequent improvement in ulcerative lesions, which was confirmed in the present study.

On the other hand, the genesis of ulcer requires acid, peptic activity and breakdown of mucosal defense mechanism [48]. However, rosiglitazone failed to produce any significant change in gastric acidity or pepsin activity compared to non-treated IND group suggesting that the gastroprotective effect of rosiglitazone does not involve attenuation of aggressive factors, rather, it acts on strengthening the defensive factors such as NO, PGE_2_ and mucus barrier.

In conclusion, rosiglitazone protects against IND-induced ulcer and this effect appears to be multifactorial. The mechanisms of this protective effect include its ability to increase NO, PGE_2_ as well as mucus secretion. In addition, the antioxidant properties of rosiglitazone seem to play a crucial role in the gastroprotection via scavenging free radicals. Thus, this study considers rosiglitazone as a more relevant anti-diabetic therapy for patients who are at risk of gastric ulcers that were induced by the frequent use NSAIDs. Thus, rosiglitazone could provide an extra benefit for patients taking NSAIDs and at high risk of developing gastric ulceration.
